# Perceived stress and mobile phone addiction among Chinese undergraduate nursing students: the mediating role of organizational caring climate and self-control

**DOI:** 10.3389/fpsyg.2026.1634642

**Published:** 2026-02-13

**Authors:** Juan Liang, Xiumin Yin, Xinting Wei, Xinyi Yang, Yanbo Ji

**Affiliations:** 1 Department of Pediatrics, The First Affiliated Hospital of Air Force Medical University, Xi’an, China; 2 Department of Thoracic Surgery, Shandong Provincial Hospital Affiliated to Shandong First Medical University, Jinan, China; 3 School of Nursing, Shaanxi University of Chinese Medicine, Xi’an, China; 4 Xi’an Jiaotong University City College, Xi’an, China; 5 Department of Nursing, The First Affiliated Hospital of Shandong First Medical University, Jinan, China

**Keywords:** mobile phone addiction, organizational caring climate, perceived stress, self-control, undergraduate nursing students

## Abstract

**Objective:**

To examine the mediating roles of organizational caring climate and self-control in the association between perceived stress and mobile phone addiction among undergraduate nursing students, and to identify potential intervention targets for reducing MPA in clinical training contexts.

**Methods:**

Between February and May 2023, a total of 900 Chinese undergraduate students from 10 colleges completed questionnaires, resulting in a response rate of 98.47%. Measures included demographic characteristics, perceived stress, organizational caring climate, self-control, and mobile phone addiction. Data were analyzed using SPSS 23.0 and the PROCESS macro.

**Results:**

Perceived stress was positively correlated with mobile phone addiction (*r* = 0.362, *P* < 0.01) and negatively correlated with organizational caring climate and self-control (*r* = -0.162 and −0.515, respectively; *P* < 0.01). Organizational caring climate was positively correlated with self-control (*r* = 0.152, *P* < 0.01) and negatively correlated with mobile phone addiction (*r* = -0.156, *P* < 0.01). Self-control was negatively correlated with mobile phone addiction (r = -0.468, *P* < 0.01). Mediation analyses indicated that perceived stress indirectly influenced mobile phone addiction through three pathways: the independent mediating effects of organizational caring climate and self-control, as well as their sequential mediating effect.

**Conclusion:**

A supportive organizational caring climate may enhance self-control among undergraduate nursing students, thereby buffering the adverse effects of perceived stress on MPA. Strengthening institutional care and individual self-regulatory capacity may be effective strategies for reducing problematic mobile phone use and promoting healthier clinical training outcomes.

## 1 Introduction

Mobile phones have become ubiquitous “digital sanctuaries” in contemporary education, particularly among students in high-demand professional disciplines such as nursing. While these devices provide convenient access to clinical and educational resources, their excessive and uncontrolled use—commonly referred to as mobile phone addiction (MPA) (Ma et al., 2021) or problematic smartphone use (Yang et al., 2020)—has emerged as a global public health concern. Systematic reviews estimate that the prevalence of MPA among nursing students ranges from 22% (Osorio-Molina et al., 2021) to 30% (Efstathiou et al., 2025) worldwide, with rates in China reported to be as high as 32.3% (Xiao et al., 2021). Notably, the COVID-19 pandemic appears to have further exacerbated this problem: prolonged periods of remote learning and social isolation substantially altered students’ digital behavior patterns, fostering greater reliance on mobile devices and increasing vulnerability to MPA (Li et al., 2022). Importantly, emerging evidence indicates that elevated levels of MPA are associated with serious mental health risks, including suicidal behaviors among Chinese adolescents, underscoring the seriousness of this behavioral problem (Cheng et al., 2024).

For undergraduate nursing students, MPA often represents a maladaptive response to the “hidden curriculum” of nursing education, which includes emotional labor, high-stakes clinical decision-making, and intensive academic workloads (Ellaway, 2014). Excessive reliance on mobile phones may reflect an imbalance in digital life balance, defined as the harmonious integration of online and offline activities, whereby digital technology is used as a compensatory strategy to escape real-world stressors (Duradoni et al., 2022). Such compensatory use can undermine this balance and contribute to a range of adverse outcomes. Accumulating evidence indicates that MPA is associated with impaired social functioning, physical discomfort (Ozdil et al., 2022), loneliness (Sönmez et al., 2021), anxiety, depression, sleep disturbances (Li et al., 2020), reduced academic performance (Rathakrishnan et al., 2021), and disordered eating behaviors (Li H. et al., 2025), ultimately hindering nursing students’ personal and professional development. Importantly, the development of MPA cannot be adequately explained by isolated factors. Instead, it should be understood as a dynamic and multifactorial process arising from the interplay between individual psychological vulnerabilities, environmental and social support systems, and the specific stressors inherent in nursing education. Consequently, a systematic examination of key risk factors and underlying mechanisms is crucial for informing theory-driven and evidence-based prevention and intervention strategies.

### 1.1 Perceived stress and mobile phone addiction

Perceived stress (PS) refers to individuals’ psychological responses to their subjective appraisal of environmental demands as threatening or overwhelming, which can adversely affect psychological wellbeing (Zheng et al., 2022). According to the Compensatory Internet Use Model, individuals experiencing chronic stress or unmet psychological needs are more likely to engage in online activities to regulate negative emotions or escape aversive realities (Kardefelt-Winther, 2014). In the context of nursing education—where the prevalence of high stress has been reported to reach approximately 62%—mobile phones offer an easily accessible, low-effort, and immediately rewarding coping outlet (Zheng et al., 2022). Accordingly, sustained exposure to stress may increase undergraduate nursing students’ vulnerability to maladaptive mobile phone use and the development of addictive patterns (Li M. et al., 2025). Based on this theoretical and empirical evidence, the present study proposes the following hypothesis:

*H1:* Perceived stress is positively associated with mobile phone addiction among undergraduate nursing students.

### 1.2 Perceived organizational caring climate as a mediator

Although the direct association between perceived stress and MPA has been well documented, clarifying the underlying psychological mechanisms remains critical for informing targeted interventions. Perceived organizational caring climate (OCC) refers to nursing students’ perceptions of the support, care, and recognition provided by nursing management staff (e.g., head nurses and clinical preceptors) during clinical placements, as well as the attentiveness, concern, and understanding demonstrated by teachers and counselors throughout their academic training, which collectively foster a sense of self-worth (Gunawan et al., 2022; Honkavuo, 2020).

Drawing on Conservation of Resources theory (Hobfoll, 1989), individuals exposed to high levels of stress experience progressive depletion of internal resources, which constrains cognitive flexibility, heightens threat sensitivity, and biases perceptions of the environment. As a result, stressed individuals may be less capable of identifying and engaging with external supportive resources, leading to more negative evaluations of their organizational context. Within this framework, OCC functions as a critical external resource. From the perspective of Self-Determination Theory, individuals possess three basic psychological needs: autonomy (volition), competence (mastery), and relatedness (belonging) (Ryan and Deci, 2000). A supportive and caring organizational climate satisfies these needs, thereby fostering high-quality autonomous motivation and reducing need frustration that often drives compensatory digital behaviors. Empirical evidence further suggests that a positive and caring climate, as a form of social support, can buffer stress, alleviate loneliness, and fill emotional deficits that underlie addictive mobile phone use as a coping strategy (Nowak et al., 2022; Unal et al., 2022; Zhang et al., 2023; Zhong et al., 2025). Based on these theoretical and empirical considerations, this study proposes the following hypotheses:

*H2:* The perceived organizational caring climate mediates the relationship between perceived stress and MPA among undergraduate nursing students. *H2a:* Perceived stress is negatively associated with perceived organizational caring climate.

*H2b*: Perceived organizational caring climate is negatively associated with MPA.

### 1.3 Self-control as a mediator

Self-control (SC) is defined as the capacity to resist external temptations and regulate behavior in accordance with internal goals (Tangney et al., 2004). A growing body of research indicates that perceived stress exerts a significant negative effect on self-control. Elevated stress is often accompanied by negative emotional states, such as anxiety and depression, which disrupt attentional focus and cognitive functioning, thereby undermining individuals’ self-regulatory capacity (He et al., 2025). According to the Strength Model of Self-Control, self-control operates as a finite resource that can be depleted through sustained exertion (Muraven et al., 2002). Managing the emotional distress and cognitive demands associated with high perceived stress consumes these limited resources, leaving individuals more susceptible to maladaptive coping strategies, including excessive mobile phone use (Duckworth et al., 2012). In contrast, individuals with higher levels of self-control are better able to resist the immediate gratification afforded by mobile phones and remain oriented toward long-term goals (Juruena et al., 2020). Consistent with this framework, empirical evidence has consistently identified self-control as a protective factor against MPA, with higher self-control associated with lower levels of impulsive and compulsive phone use (Geng et al., 2021; Pan et al., 2023; Peng et al., 2022). Taken together, these findings suggest that self-control may function as a key psychological mechanism linking perceived stress to MPA. Accordingly, the present study proposes the following hypotheses:

*H3:* Self-control mediates the relationship between perceived stress and MPA.

*H3a:* Perceived stress is negatively associated with self-control.

*H3b:* Self-control is negatively associated with MPA.

Empirical evidence indicates that individuals who perceive higher levels of organizational care experience less emotional exhaustion and maintain greater self-regulatory capacity (Larsman et al., 2024; Ren et al., 2024). In addition, social support has been shown to enhance self-control, particularly among individuals with a lower sense of meaning in life (Lin et al., 2025; Liu et al., 2022). Integrating these findings with Conservation of Resources and Self-Determination theories, a supportive organizational caring climate may buffer stress and fulfill individuals’ basic psychological needs by fostering group identity and self-confidence. In turn, this process strengthens students’ self-control and reduces reliance on compensatory digital behaviors. Accordingly, the present study proposes the following hypotheses:

*H4:* Perceived organizational caring climate and self-control serially mediate the relationship between perceived stress and MPA.

*H4a:* Perceived organizational caring climate is positively associated with self-control.

Although prior research has independently linked stress, self-control, and social support to MPA, a critical gap remains in understanding the dynamic interaction between environmental resources—such as organizational caring climate —and internal regulatory mechanisms, including self-control. To address this gap, the present study integrates Compensatory Internet Use Model, Conservation of Resources theory, and the Strength Model of Self-Control into a unified conceptual framework to elucidate the serial pathway from perceived stress to mobile phone addiction.

## 2 Materials and methods

### 2.1 Participants and procedures

A multi-stage cluster-stratified random sampling method was employed to recruit 4-year undergraduate nursing students from 10 schools in Shanxi Province, Northwest China, between February to May 2023. The sampling process was as follows: (1) one undergraduate medical college was randomly selected from each of the 10 cities in the province; (2) students within each college were stratified by grade (freshman to senior); (3) one class was randomly selected from each grade; and (4) approximately 40–50% of students within each selected class were randomly invited to participate. Students on a leave of absence, those enlisted in active military service, or individuals who had not completed at least 6 months of their current academic or clinical phase were excluded.

The sample size was calculated using a multistage cluster-stratified random sampling calculation formula, based on a prior study’s reported MPA prevalence of 25.1% among Chinese nursing students (Wang et al., 2020), so *P* = 25.1%. A design effect (*Deff*) was set to 1.5 with a two-tailed α of 0.05 (*u* = 1.96) and an allowable error (*d*) set at 0.15*P*. The sample size was estimated using the following equation:


$$N=D\,e\,f\,f\,\frac{u^{2}\,P\,\left(1-P\right)}{d^{2}}.$$

This yielded a minimum required sample size of 764. After accounting for an anticipated attrition rate of 15%, the target sample size was adjusted to 878. A total of 914 questionnaires were distributed by trained researchers. After excluding 14 questionnaires due to irregular response patterns or insufficient completion time, 900 valid questionnaires were retained, resulting in an effective response rate of 98.47%. The retained questionnaires were fully completed, with no missing data. This sample size provided adequate statistical power ( > 0.80) to detect small-to-moderate indirect effects in serial mediation analyses. Although recruitment across multiple institutions enhanced the generalizability of the findings, potential cluster-level biasesecruitas differences in curricula and clinical placement intensity—should be considered. To partially control for potential cluster-level variations, gender and academic grade were included as covariates in all regression and mediation analyses, thereby adjusting for differences in curricula and clinical intensities across the 10 colleges.

The study was conducted in accordance with the Declaration of Helsinki and was approved by the Research Ethics Committee of Tangdu Hospital (Approval ID: TDLL-202210-17). Informed consent was obtained from all participating schools, teachers, and students prior to data collection. Participants were assured of the anonymity and confidentiality of their responses and their right to withdraw at any time.

### 2.2 Measures

Perceived stress was assessed using the 14-item Chinese version of the Perceived Stress Scale (PSS-14, Yang and Huang, 2003), originally developed by Katsarou et al. (2012) and widely validated in Chinese populations. The scale is particularly appropriate for nursing students, as it assesses the subjective perception of life situations as unpredictable or uncontrollablen of life rception of life sinto the hidden curriculum of nursing education. It comprises two dimensions: sense of loss of control (7 items) and sense of tension (7 items). Importantly, this conceptualization aligns with the Strength Model of Self-Control, in which sustained tension contributes to cognitive resource depletion and weakened self-regulatory capacity. Items are rated on a 5-point Likert scale ranging from 1 (om 1 rangto 5 (1 ranging ngingseven items reverse-scored. Total scores range from 14 to 70, with higher scores indicating greater perceived stress. Previous research has demonstrated good reliability and validity of the scale (Meng et al., 2022). In the present study, the PSS-14 showed acceptable internal consistency (Cronbach’s α = 0.741).

Organizational caring climate was measured using the Organizational Caring Climate Questionnaire (OCCQ), originally developed by Hughes et al.,(Hughes et al., 2016), emphasizing humanistic interactions within educational and clinical environments. The Chinese version was revised by Chen (2017) to investigate the level of perceived organizational caring climate among undergraduate nursing students and internship nursing students. The OCCQ consists of three dimensions: exemplar (14 items), dialogue (9 items), and affirmation (7 items)—which are conceptually aligned with Self-Determination Theory. Exemplar (mentors’ role modeling) and dialogue (open communication) support students’ needs for relatedness and competence during clinical placements, whereas affirmation (recognition of effort) functions as a critical external resource that buffers the “resource loss spiral” described in Conservation of Resources theory. Together, these dimensions capture the external resource replenishment mechanism hypothesized to sustain students’ internal self-regulatory capacity. Items are rated on a 6-point Likert scale from 1 (“strongly disagree”) to 6 (“strongly agree”). Total scores range from 30 to 180, with higher scores reflecting a stronger perceived caring climate. The scale has demonstrated excellent psychometric properties in prior studies (Li S. et al., 2021). In the current study, internal consistency was excellent (Cronbach’s α = 0.971).

Self-control was assessed using the Chinese version of the Self-Control Scale (Tangney et al., 2004) adapted by Tan and Guo (2008). The scale measures individuals’ capacity to regulate impulses and pursue long-term goals and includes 19 items across five dimensions: impulse control (6 items), health habits (3 items), resistance to temptation (4 items), work focus (3 items), and moderation of entertainment (3 items). Conceptually, its multidimensional structure—particularly impulse control and resistance to temptation—maps directly onto behavioral manifestations of ego depletion in digital contexts. Given the cognitively demanding nature of clinical training, the work focus dimension is especially relevant for examining how stress-related regulatory failure translates into maladaptive coping behaviors. Items are rated on a 5-point Likert scale ranging from 1 (“completely disagree”) to 5 (“completely agree”), with several items reverse-scored. Total scores range from 19 to 95, with higher scores indicating greater self-control. The scale has shown strong reliability and validity in previous research (Du and Zhang, 2022). In this study, Cronbach’s α was 0.879.

Mobile phone addiction was assessed using the Mobile Phone Addiction Tendency Scale (MPATS), a multidimensional measure of behavioral dependency developed for Chinese university students (Jie et al., 2012). The 16-item scale assesses problematic mobile phone use across four dimensions: withdrawal symptoms (6 items), salience (4 items), social comfort (3 items), and mood modification (3 items). These dimensions align with the Compensatory Internet Use Model, with social comfort and mood modification being particularly relevant for understanding how students use mobile devices as “digital sanctuaries” to cope with clinical stressors. Items are rated on a 5-point Likert scale from 1 (“strongly disagree”) to 5 (“strongly agree”). Total scores range from 16 to 80, with scores ≥ 48 indicating mobile phone addiction; higher scores reflect greater addiction severity. The MPATS has demonstrated good reliability and validity in prior studies (Han et al., 2023; Zhang et al., 2022). In the present study, the scale showed excellent internal consistency (Cronbach’s α = 0.927).

All measures employed validated Chinese versions. For scales originally developed in English (PSS-14, OCCQ, and Self-Control Scale), standard forward- and back-translation procedures were followed during prior adaptations to ensure linguistic and conceptual equivalence.

Data regarding sex (male/female) and grade (freshman/sophomore/junior/ senior) were obtained for each participant.

### 2.3 Data analysis

Data were analyzed using IBM SPSS Statistics 23.0 and the PROCESS macro (v4.0) for SPSS. Descriptive statistics and Pearson’s correlation analysis were used to summarize the data and examine bivariate relationships between variables. To test the hypothesized serial mediation model, we used Model 6 of the Hayes’ PROCESS macro. This model estimates the direct association of perceived stress (X) with mobile phone addiction (Y), as well as the indirect associations through perceived organizational caring climate (M1) and self-control (M2) in a serial causal chain (X → M1 → M2 → Y). Gender and grade were included as covariates in all regression models. The significance of indirect effects was determined using bootstrapping with 10,000 resamples to generate bias-corrected 95% CI. An indirect effect is considered statistically significant if its 95% CI does not contain zero. Prior to the main analysis, checks for the assumptions of linear regression were conducted. No major violations of linearity, independence of errors, or normality of residuals were detected. Multicollinearity diagnostics were also performed; Variance Inflation Factor (VIF) scores for all predictors were below 1.5, indicating that multicollinearity was not a concern.

## 3 Results

### 3.1 Preliminary analyses

As presented in Table 1, of the 900 participants, 94% were female. The distribution across academic grades was: freshmen (*n* = 195, 21.7%), sophomores (*n* = 206, 22.9%), juniors (*n* = 260, 28.9%), and seniors (*n* = 239, 26.6%). Using the cutoff score of ≥ 48 on the MPATS, the prevalence of MPA in this sample was 34.9%. A one-way analysis of variance showed no significant differences in MPA based on gender or grade (*P* > 0.05).

**TABLE 1:** Demographic characteristics and comparison of MPA of undergraduate nursing students (*N* = 900).

Variable	Category	*N*(%)	Mobile phone addiction
Gender	Male	54(6)	40.94 ± 10.06
	Female	846(94)	41.49 ± 10.13
	*T*		−0.383
	*P*		0.702
Grade	Freshman	195(21.67)	41.26 ± 9.87
	Sophomore	206(22.88)	42.86 ± 8.81
	Junior	260(28.89)	41.27 ± 10.44
	Senior	239(26.56)	41.29 ± 9.92
	*F*		0.769
	*P*		0.511

### 3.2 Common method bias test

The data in this study were collected through self-reported measures, which may be susceptible to common method bias. To address this concern, anonymous responses and reverse scoring were used to control common method bias during implementation. Additionally, Harman’s one-factor method was applied to the four questionnaires to test for the presence of common method bias, and exploratory factor analysis was performed on the items related to the four research variables. Among the 79 items, 12 factors with eigenvalues > 1 were extracted. The first common factor explained 24.332% of the variance, which was less than the critical value of 40%, demonstrating that common method bias was not a problem in this study.

### 3.3 Bivariate correlation analysis

The means, standard deviations, and correlations of each variable are presented in Table 2. The results show that perceived stress was negatively correlated with organizational caring climate (*r* = -0.162, *P* < 0.01) and self-control (*r* = -0.515, *P* < 0.01), but positively correlated with mobile phone addiction (*r* = 0.362, *P* < 0.01). Organizational care climate was positively correlated with self-control (*r* = 0.152, *P* < 0.01) and negatively correlated with mobile phone addiction (*r* = -0.156, *P* < 0.01). Self-control was negatively correlated with mobile phone addiction (*r* = -0.468, *P* < 0.01).

**TABLE 2:** Descriptive statistics and Pearson’s correlations of the research variables.

Variable	M	SD	α	ω	1	2	3	4
Perceived stress	40.07	5.90	0.741	0.805	1	1	1	1
Organizational caring climate	128.14	27.21	0.971	0.971	−0.162**			
Self-control	61.71	9.96	0.879	0.884	−0.515**	0.152**		
Mobile phone addiction	41.46	10.12	0.927	0.923	0.362**	−0.156**	−0.468**	

**P < 0.01.

### 3.4 Test the mediating effect of organizational caring climate and self-control in perceived stress and mobile phone addiction

**FIGURE 1:**
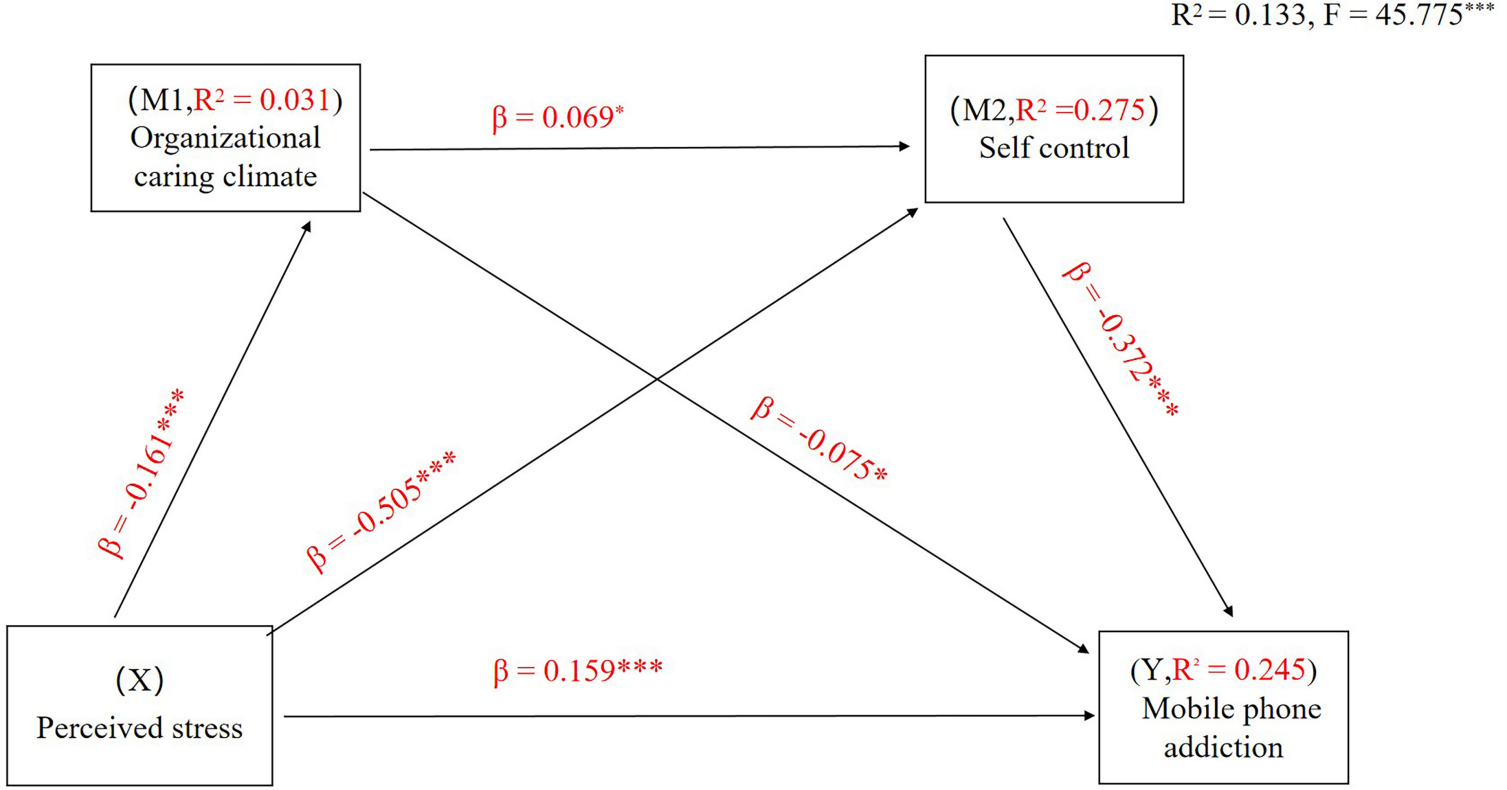
Serial-multiple mediation of organizational caring climate and self-control in the relationship between perceived stress and mobile phone addiction with standardized beta values. ***P < 0.001.

The mediating effect of organizational caring climate and self-control on the relationship between perceived stress and MPA was determined. The results of the analysis of perceived stress on MPA mediated by organizational caring climate and self-control are presented in Figure 1 and Table 3. The whole regression equation was significant (*R*^2^ = 0.133, *F* = 45.775, *P* < 0.001).

**TABLE 3:** Multiple linear regression analysis of the variables.

Regression model		Overall fit index			Significance of regression coefficients		
Dependent variable	Independent variable	*R*	*R ^2^*	*F*	β	β (standardization)	t
Organizational caring climate		0.177	0.031	9.666			
	Gender				8.373	0.064	2.183*
	Grade				−0.738	0.026	−0.679
	Perceived stress				−0.736	−0.161	−4.844***
Self-control		0.524	0.275	84.830			
	Gender				−1.048	−0.025	−0.860
	Grade				0.797	0.078	2.311*
	Organizational caring climate				0.027	0.069	2.518*
	Perceived stress				−0.856	−0.505	−17.565***
Mobile phone addiction		0.495	0.245	57.900			
	Gender				0.989	0.026	0.782
	Grade				−0.194	−0.034	−0.539
	Organizational caring climate				−0.028	−0.075	−2.559*
	Self-control				−0.380	−0.372	−10.956***
	Perceived stress				0.271	0.159	4.627***

*P < 0.05,

***P < 0.001. All variables have VIF values < 1.5, indicating no multicollinearity issues.

The total effect of perceived stress on MPA was found to be significant (c = 0.625, *t* = 11.695, *P* < 0.001) (Step 1). In addition, perceived stress had a negative direct effect on organizational caring climate (β = -0.161, *t* = -4.844, *P* < 0.001) and self-control (β = -0.505, *t* = -17.565, *P* < 0.001). The direct effect of organizational caring climate as the first mediating variable on the second mediating variable of self-control was also significant (β = 0.069, *t* = 2.518, *P* < 0.05) (Step 2). A review of the direct effects of the mediating variables on MPA showed that the effects of an organizational caring climate (β = -0.075, *t* = -2.559, *P* < 0.05) and self-control (β = -0.372, *t* = -10.956, *P* < 0.001) were significant (Step 3). When perceived stress and the two mediating variables were simultaneously entered into the model (Step 4), the direct effect of perceived stress (c’ = 0.271, *t* = 4.627, *P* < 0.001) on MPA were also found to be significant. Overall, these results revealed that multiple serial mediations had occurred. Notably, the effect of self-control far outweighs that of the organizational climate, suggesting that while the environment provides the resources, internal regulation remains the proximal driver of addictive behavior.

As presented in Table 4, when considering all variables (including covariates) in the tested model, the paths through single mediation of organizational care climate [point estimate = 0.012; 95% BC CI (0.001, 0.024)], self-control [point estimate = 0.189; 95% BC CI (0.140,0.240)], and both mediators [point estimate = 0.004; 95% BC CI (0.001,0.010)] were all statistically significant. The total indirect effect was statistically significant [point estimate = 0.201; 95% confidence interval (0.156,0.256)]. Thus, the path through both mediators was significant, and the indirect effect through both organizational caring climate and self-control was also significant.

**TABLE 4:** Comparison of indirect effects of perceived stress on mobile phone addiction mediated by organizational caring climate and self-control.

Path	Point estimate	Boot SE	BootLL CI	BootUL CI	Relative effect value
Total indirect effect of X on Y	0.201	0.025	0.156	0.256	56.78%
Indirect effect 1: X → M1 → Y	0.012	0.006	0.001	0.024	3.31%
Indirect effect2: X → M2 → Y	0.189	0.025	0.140	0.240	52.30%
Indirect effect3: X → M1 → M2 → Y	0.004	0.002	0.001	0.010	1.19%
Model1 versus model 2	−0.177	0.026	−0.230	−0.127	
Model1 versus model 3	0.008	0.007	−0.006	0.021	
Model2 versus model 3	0.185	0.024	0.140	0.234	

X, perceived stress; M1, organizational caring climate; M2, self-control; Y, mobile phone addiction; Model 1, perceived stress- organizational caring climate- mobile phone addiction; Model 2, perceived stress- self-control- mobile phone addiction; Model 3, perceived stress- organizational caring climate- self-control- mobile phone addiction; LL, lower level; UL, upper level.

## 4 Discussion

The present study examined the psychological mechanisms through which perceived stress influences MPA among Chinese undergraduate nursing students and empirically validated a serial mediation model involving organizational caring climate and self-control. The findings demonstrate that the association between stress and MPA is not merely direct but is fundamentally shaped by the interaction between environmental resources within the educational context and students’ internal self-regulatory capacity, offering a comprehensive explanation of how digital behaviors become dysregulated in high-pressure clinical education settings.

### 4.1 Impact of perceived stress on mobile phone addiction

The direct impact of perceived stress on MPA remains a critical finding, confirming Hypothesis 1. Nursing students are exposed to the distinctive pressures of the “hidden curriculum,” including emotional labor, high-stakes clinical decision-making, and intensive academic demands (Hosseini et al., 2023; Li et al., 2024). Under these conditions, mobile phones are often used as “digital sanctuaries.” This behavior is effectively explained by the Compensatory Internet Use Model, which posits that individuals engage in online activities to escape real-world stressors or fulfill unmet psychological needs (Chen and Chang, 2019).

Extending beyond this direct association, our findings resonate with the Digital Life Balance framework, which conceptualizes problematic mobile phone use as a dynamic process of disharmonization between digital and offline domains rather than a simple failure of self-discipline (Duradoni et al., 2024). In high-pressure nursing programs, digital technology often serves as a primary coping tool to alleviate emotional discomfort or fulfill unmet needs for relatedness and control. According to theories of harmony and harmonization (Di Fabio and Tsuda, 2018), chronic stress may push students toward digital environments to compensate for frustrations experienced in real-life contexts. However, excessive reliance on digital coping can result in temporal displacement, whereby essential restorative activitiesnce onas sleep, physical exercise, and meaningful face-to-face interactionsgful facecn digital coping can re-based engagement (Duradoni et al., 2025). From this perspective, MPA represents a maladaptive attempt to restore psychological equilibrium under sustained stress, ultimately undermining students’ holistic wellbeing. Accordingly, effective interventions should target the underlying imbalance between digital and real-life resources rather than focusing solely on restrictive or punitive measures.

### 4.2 Mediating role of organizational caring climate in the relationship between perceived stress and MPA

The mediating role of OCC elucidates the environmental mechanisms underlying MPA, thereby supporting Hypothesis 2 and highlighting the role of contextual resources in shaping maladaptive digital behaviors. Integrating Conservation of Resources theory with Self-Determination Theory, OCC represents a salient external resource that buffers stress-related resource depletion while supporting the fulfillment of autonomy, competence, and relatedness needs. Within caring educational environmentss.lment of aurnal resonservation of ResMPA, thereby supporting Hypotision, and a culture of recognitionnmentss.lment of aurnal resonservation of ResMPA, thereby supporting Hypotto excessive mobile phone use as a compensatory strategy (Del Prato et al., 2011). Conversely, elevated perceived stress may precipitate a resource loss spiral, weakening students’ perception of available organizational support and increasing reliance on mobile devices to compensate for unmet psychological needs, particularly those related to control and social belonging (Hobfoll et al., 2018).

Accordingly, a strong caring climate enhances autonomous motivation, mitigates need frustration linked to addictive behaviors, and facilitates digital life balance by encouraging meaningful offline interactions. This interpretation aligns with Honkavuo (2020) findings among Finnish nursing students, which underscore the developmental value of caring relationships grounded in mutual respect and inquiry-based pedagogy (Honkavuo, 2020). Empirical evidence further indicates that perceptions of organizational care are associated with reduced work withdrawal and enhanced self-efficacy (Gao et al., 2026; Larsman et al., 2024). By offering psychologically safe spaces for expression and affirming students associated with reduced work withdrawal and enhanced self-efficacy (Gao et al., 2026; Larsman et al., 2024). By offering psychologically safe spaces for expression and affirming students’ clinical contributions, organizations strengthen students’ sense of mattering, thereby diminishing their tendency to seek rapid external validation through mobile phone–mediated interactions (e.g., social media engagement).

### 4.3 Mediating role of self-control in the relationship between perceived stress and MPA

The mediating role of self-control corroborates the Strength Model of Self-Control and provides support for Hypothesis 3. Self-control represents a finite regulatory resource that is progressively depleted by the sustained effort required to manage negative emotions and high-stakes clinical demands. Consistent with the ego depletion framework, our findings indicate that perceived stress exerts a substantial negative effect on self-control. When nursing students experience emotional exhaustion, their capacity to resist immediately rewarding digital stimuli—such as social media, gaming, or short-form video content—is markedly diminished.

This breakdown in self-regulation appears to be the most proximal mechanism driving mobile phone addiction. Notably, the indirect effect of self-control accounted for 52.30% of the total effect, substantially exceeding the indirect contribution of organizational caring climate (3.31%). These results underscore that although supportive environments provide essential contextual resources, the preservation of internal self-regulatory capacity is the most critical factor in preventing addictive mobile phone use.

### 4.4 Sequential mediation model

Crucially, the serial mediation pathway (Stress → OCC → SC → MPA) indicates that environmental and internal resources function not as independent buffers but as interdependent layers of resource protection. A caring organizational climate operates as a replenishment mechanism for self-control by providing emotional support and professional guidance. Such support reduces students’ cognitive and emotional load, thereby slowing ego depletion and preserving the self-regulatory capacity required to maintain a healthy relationship with digital technology. This sequential process underscores that institutional care constitutes a foundational condition for effective individual self-regulation in high-stress vocational training contexts.

More specifically, negative affective responses triggered by perceived stress—such as anxiety and depressive symptoms—can disrupt attentional focus and cognitive processing, leading to impairments in inhibitory control mediated by the prefrontal cortex (He et al., 2025; Li H. et al., 2021; Li H. et al., 2025). Under conditions of combined psychological and physiological depletion, nursing students become more susceptible to impulsive decision-making and are more likely to prioritize low-effort, immediately rewarding behaviors, such as mobile phone use. When the external environment simultaneously lacks sufficient affirmation and emotional containment, students may experience heightened helplessness, further reinforcing reliance on digital compensation.

Together, these findings suggest that interventions targeting mobile phone addiction should place self-control training at their core, while simultaneously enhancing organizational caring climates to optimize environmental support and prevent downstream self-regulatory failure.

### 4.5 Limitations and implications

Several limitations should be acknowledged. First, the cross-sectional design precludes causal inference; future research should adopt longitudinal approaches to examine cycles of resource depletion and replenishment over extended periods (e.g., 12–24 months). Second, reliance on self-report measures may introduce social desirability bias, although diagnostic tests indicated no serious concern in the present study. Third, the sample was drawn from a single province in China, which may limit the generalizability of findings to nursing students in other cultural or educational contexts. Future research should test the robustness of this model among students from other healthcare disciplines (e.g., medicine, pharmacy, public health) and in different cultural or educational systems to determine the universality of the observed mechanism. Lastly, although the proposed model accounted for 24.5% of the variance in MPA, future studies should incorporate a broader range of individual-, family-, and organizational-level factorsvel faas internship workload intensity, family-level stressors, localized hospital management styles, variability in humanistic care training, dispositional mindfulness, physical activity habits (Tian et al., 2025), personality traits (e.g., neuroticism and trait mindfulness; Duradoni et al., 2025; Lane et al., 2021), and coping styles to develop a more comprehensive understanding of MPA in this population.

Despite these limitations, the findings provide a robust empirical basis for proactive, multi-level interventions aimed at restoring digital life balance and mitigating the adverse effects of digital dependency. At the individual level, nursing programs should integrate skills-based self-control training into the curriculum. Brief, online mindfulness-based interventions have demonstrated efficacy in reducing MPA by enhancing attentional control and diminishing impulsive responding (Priyadarsini et al., 2024; Sumińska and Rynkiewicz, 2025). Notably, emerging evidence indicates that trait mindfulness exerts a protective psychological effect by moderating the relationship between digital life balance and MPA (Aldbyani et al., 2025), thereby identifying a critical and malleable skills-based target for self-regulation training in nursing education. In addition, workshops centered on implementation intentions (e.g., “if–then” plans for mobile phone use) may equip students with practical strategies to manage digital temptations during clinical placements (Olson et al., 2022; Tang and Lee, 2021). These interventions, which often yield moderate-to-large effect sizes (Cohen’s *d* ≈ 0.5), are particularly feasible when delivered via mobile platforms, offering accessible and stigma-free support.

At the organizational level, nursing schools and clinical institutions should prioritize cultivating a culture of care. Training faculty members and head nurses in humanistic care and empathetic leadership may enhance students’ perceptions of organizational support (Yang et al., 2024). Institutions may also implement structural policies that promote digital life balance, such as establishing phone-free recovery spaces and encouraging offline social engagement and physical activity (Lantz and Fagefors, 2025). Furthermore, emerging AI-assisted, personalized stress-management tools—such as adaptive mindfulness exercises or reflective writing interventions—hold promise for delivering timely and tailored support based on students’ fluctuating levels of stress and self-regulatory depletion (Tian et al., 2025).

## 5 Conclusion

In conclusion, this study reveals that perceived stress influences MPA through a complex serial mediation involving the depletion of both environmental (OCC) and internal (SC) resources. By framing MPA as a compensatory attempt to restore digital life balance, we provide a new theoretical direction for intervention. Enhancing institutional support and individual regulatory capacity are both essential for mitigating the negative impacts of digital dependency on the mental health and professional development of future nursing professionals.

## Data Availability

The original contributions presented in the study are included in the article/supplementary material, further inquiries can be directed to the corresponding author.
